# An improved chromosome-level genome assembly and annotation of *Echeneis naucrates*

**DOI:** 10.1038/s41597-024-03309-w

**Published:** 2024-05-04

**Authors:** Tianxiang Gao, Kai Liu, Qi Liu, Danyang Wang

**Affiliations:** 1 Fisheries College, Jimei University, Xiamen, 361021 China; 2 Wuhan Onemore-tech Co., Ltd, Wuhan, 430000 China; 3 MOE Key Laboratory of Marine Genetics and Breeding, College of Marine Life Sciences, Ocean University of China, Qingdao, 266100 China

**Keywords:** Genome, Agricultural genetics

## Abstract

*Echeneis naucrates*, as known as live sharksucker, is famous for the behavior of attaching to hosts using a highly modified dorsal fin with oval-shaped sucking disc. Here, we generated an improved high-quality chromosome-level genome assembly of *E. naucrates* using Illumina short reads, PacBio long reads and Hi-C data. Our assembled genome spans 572.85 Mb with a contig N50 of 23.19 Mb and is positioned to 24 pseudo-chromosomes. Additionally, at least one telomere was identified for 23 out of 24 chromosomes. Furthermore, we identified a total of 22,161 protein-coding genes, of which 21,402 genes (96.9%) were annotated successfully with functions. The combination of *ab initio* predictions and Repbase-based searches revealed that 15.57% of the assembled *E. naucrates* genome was identified as repetitive sequences. The completeness of the genome assembly and the gene annotation were estimated to be 97.5% and 95.4% with BUSCO analyses. This work enhances the utility of the live sharksucker genome and provides a valuable groundwork for the future study of genomics, biology and adaptive evolution in this species.

## Background & Summary

**Fig. 1:**
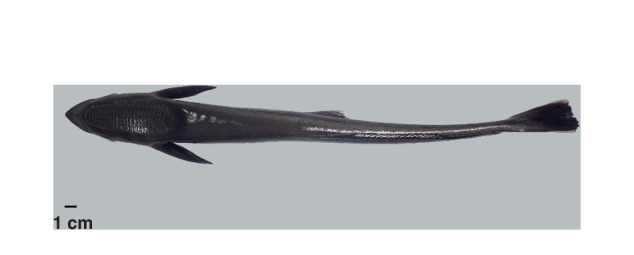
Morphological characteristics of *E. naucrates*.

Live sharksucker (*Echeneis naucrates*), also known as the sluggard in the ocean, is in the Echeneidae family, order Carangiformes (Fig. 1). This sharksucker is widely found in tropical and warm temperate waters^1^, and ranging from coastal areas to those offshore^2^. The key distinctive characteristic to distinguish it from other fishes is the oval-shaped sucking disc, which is a highly modified dorsal fin and used to attach to hosts. The oval-shaped sucking disc comprises of 21–28 laminae and extends from the top of the head to the front part of the body^3^. The hosts of live sharksucker encompass whales, sharks, dolphins, sea turtles, divers and vessel hulls^4–7^. With a host, proposed benefits to live sharksucker comprise conveyance (via “hitchhiking”), shielding from predators, enhanced courtship and reproductive capacity, improved gill aeration and expanded feeding opportunities^8^. The unique suction cups and adsorption habits make the live sharksucker a good research subject for bionic study^9,10^, aid in fishing^11^ and adaptive evolution, such as the commensalism relation between remora fish and shark^12^. Nonetheless, our comprehension of the biological context of the live sharksucker remains constrained.

Genome sequencing has played a pivotal role in advancing various aspects of basic biology. High-quality reference genomes could profoundly enhance our understanding of the genetic foundation and the evolutionary process underlying unique biological characteristics in the live sharksucker. Although the chromosome-level live sharksucker genome has been released on NCBI with GenBank assembly accession GCA_900963305.1^13,14^ and GCA_900963305.2^15^, the completeness of genome assembly and annotations still require further refinement. For instance, the released chromosome-level genome assembly remained incomplete with many gaps (average 110.13 N’s per 100 kbp) (Fig. 3b). Not only that, a number of annotation details, including information related to repeats and non-coding RNAs, have not been made publicly available and remain inaccessible.

**Fig. 2:**
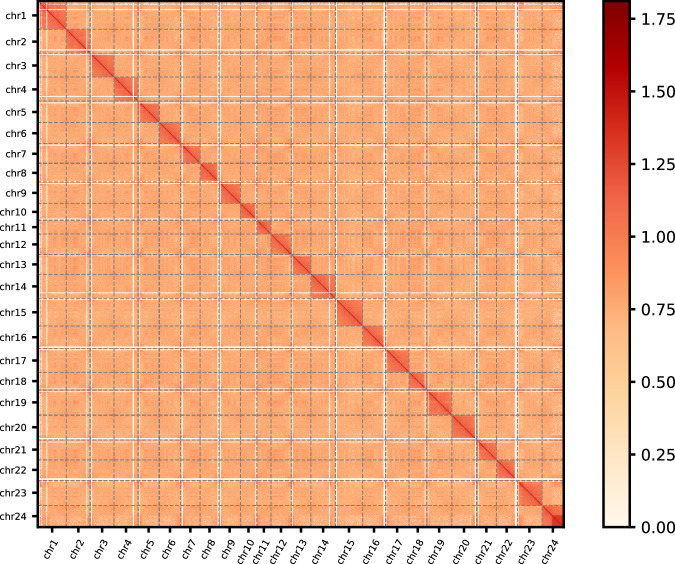
Hi-C interaction heat map for genome assembly of *E. naucrates*. The interaction density is quantified based on the number of supporting Hi-C reads and depicted using a color gradient ranging from white (low density) to dark red (high density).

**Fig. 3:**
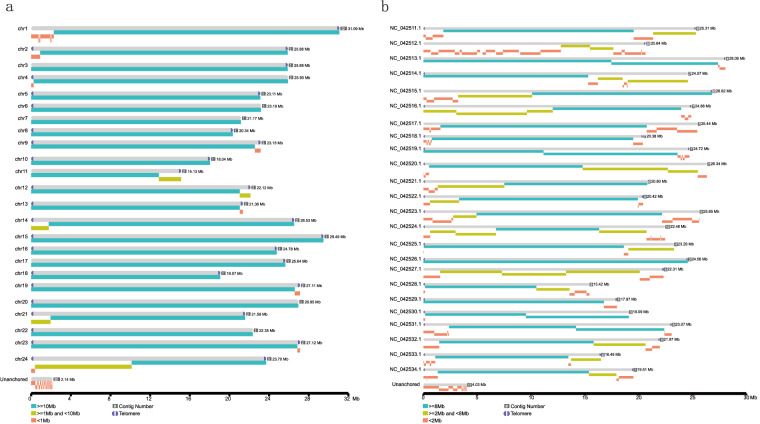
Comparison of genome assembly of *E. naucrates* with the previous version. Contig distribution maps for chromosomes of *E. naucrates* between the assembly (**a**) in this study and (**b**) the previous version. The bars in grey represent entire lengths of chromosomes, in which the positions of telomeres are shown. The contig numbers and the sizes of chromosomes were shown behind the bars.

In this study, we generated 33.14 Gb of PacBio High fidelity (HiFi) long-reads with the N50 length of 18.11 kb, and 89.93 Gb of Illumina paired-end sequencing short-reads for genome assembly (Table 1). An additional 76.64 Gb of high-throughput chromatin capture (Hi-C) sequencing data were utilized to validate the genome assembly through a comparison with the scaffolding data. Leveraging these integrated sequencing data, we constructed a high-quality chromosome-level reference genome of *E. naucrates*. Specifically, a 572.85 Mb genome was assembled, comprised of 54 contigs with the contig N50 length of 23.19 Mb. A total of 570.71 Mb (99.63% of the contig-level genome) of the assembled sequences were positioned to 24 pseudo-chromosomes with low missing bases (average 0.40 N’s per 100 kbp). Moreover, telomeres were identified for at least one end of 23 out of 24 chromosomes, totaling 38 telomeres (Fig. 3a and Table 7). In this enhanced genome assembly, we have improved upon previous gene annotations by amalgamating *ab initio* predictions, protein homology searches and transcriptome-assisted methods, which identified a total of 22,161 protein-coding genes. Through a dual approach involving both homology searches and ab initio predictions, 15.57% of the assembled *E. naucrates* genome was identified as repetitive sequences. BUSCO alignment analysis of assembly based on the actinopterygii_odb10 database revealed that our ultimate assembly encompassed 3, 551 (97.5%) complete BUSCOs. The consensus QV of genome assembly was 52.01. In summary, this high-quality chromosome-level reference genome serves as a valuable foundation for the utilization of genetic resources, and the further investigation of the unique biological characteristics, such as the oval-shaped sucking disc, in the live sharksucker.

**Table 1:** Statistics of sequencing data for *E. naucrates* genome assembly and annotation.

Library type	Tissue	Raw data (Gb)	Clean data (Gb)	Average read length (bp)
WGS Illumina	Muscle	89.93	87.77	150
PacBio HiFi	Muscle	495.96	33.14	17,897
Hi-C	Muscle	76.64	76.56	150
RNA-seq	Pooled	33.72	33.01	150

**Table 2:** Comparison of *E. naucrates* genome assembly metrics with previous version.

	*E. naucrates*	
	This study	fEcheNa1.1/fEcheNa1.2
Sequenced genome size (Mb)	572.85	544.2
Contig N50 (Mb)	23.19	12.4
Scaffold N50 (Mb)	24.78	23.3
Gap size (N’s per 100 kbp)	0.40	110.1
Complete BUSCOs (%)	97.86	99.1
Fragmented BUSCOs (%)	0.38	0.2
Missing BUSCOs (%)	1.76	0.7
Duplicated BUSCOs (%)	0.82	1.2

## Methods

### Sample collection and preparation

A single fish (~1500 g) was obtained in June 2022 from Northern South China Sea. The sampled fish in this study was permitted by the Animal Care and Use Committee of Fisheries College of Jimei University (Animal Ethics no. 1067) and performed by the regulations and guidelines established with this committee. Dorsal muscle, dorsal fin, skin, skull, and skull muscle tissues were collected and preserved in liquid nitrogen until the extraction of DNA and RNA. Dorsal muscle tissues were utilized for DNA sequencing to construct the genome assembly, while all tissues were utilized for RNA sequencing. The quality and quantity of genomic DNA samples were assessed through 1% agarose gel electrophoresis and the Pultton DNA/Protein Analyzer (Plextech).

### WGS Illumina library construction, sequencing and assembly

To create the whole-genome sequencing (WGS) Illumina library, a paired-end library was constructed with an insert size of 300 bp adhering to the Illumina standard protocol. Then, DNA was purified, quantified, and sequenced from both ends using the Illumina NovaSeq 6000 sequencing platform. In total, a sum of 89.93 Gb raw reads was obtained (Table 1). After filtering process by using fastp v 0.23.2^16^ with default parameters to remove low quality and short reads, as well as trim adapters and polyG sequences, a set of 87.77 Gb clean data were retained (Table 1). The estimation of the genome size and heterozygosity for live sharksucker was then performed using GCE v 1.0.0^17^ by k-mer analysis with clean Illumina short data following the default settings.

### PacBio library construction, sequencing and assembly

To obtain the PacBio long reads, a SMRTbell library was established with a fragment size of 20 kb using the SMRTBell template preparation kit 1.0 (PacBio) in accordance with the manufacturer’s instructions. The library was sequenced with the PacBio Sequel II system in Circular Consensus Sequence (CCS) mode. Upon the elimination of low-quality reads, a sum of 33.14 Gb reads with an average length of 17.90 kb were retained and then processed with the CCS v 6.0.0 algorithm with default parameters. With these PacBio long reads, the initial contigs were subsequently assembled using the Hifiasm v 0.16.1 algorithm^18^ with the default settings. After that, the purge_haplotigs v1.0.4^19^ with the parameter of ‘-a 70 -j 80 -d 200’ was employed to eliminate redundant sequences. This procedure resulted in a contig-level assembly of about 588.30 Mb comprised of 54 contigs, with the N50 and maximum contig size of 23.19 Mb and 29.49 Mb, respectively.

### Hi-C library preparation, sequencing and chromosome assembly

Hi-C data were used to anchor contigs onto chromosomes. Briefly, dorsal muscle tissue (~1 g) of *E. naucrates* was fixed with 1% formaldehyde for 10–30 min at room temperature (20–25 °C) to congeal proteins involved in chromatin interactions within the genome. DNA was digested with the 4-cutter restriction enzyme MboI. The overhangs of restriction fragments were filled and labeled with biotinylated nucleotides, followed by ligation in a compact volume. Following the cross-link reversal, the ligated DNA was purified and fragmented to a size range of 300–500 bp. Subsequently, ligation junctions were extracted by binding to streptavidin beads and prepared for Illumina NovaSeq 6000 sequencing. In total, 76.64 Gb of Hi-C reads were obtained (Table 1). After filtering reads with average quality scores less than 20 and removing adapters using fastp v 0.23.2^16^ with the default settings, a total of 76.56 Gb clean data were retained (Table 1). We also utilized the HiCUP pipeline^20^, with the parameter of ‘--re1 ^GATC,MboI’ in hicup_digester step, to remove the erroneous mappings and duplicated contigs to yield the interaction matrix. This matrix served as the foundation for anchoring the contigs onto chromosomes through the utilization of approximately 169.29 Mb read pairs (~ 68.27%) via the 3D-DNA pipeline^21^ with the default settings. The scaffolds were subjected to a manual assessment and refinement process utilizing Juicebox Assembly Tools^22^ in order to rectify any instances of chromosome translocation and inversion. By integrating this Hi-C data, the contig-level assembled sequences were positioned onto 24 pseudo-chromosomes, encompassing a cumulative length of 570.71 Mb, covering ~99.63% of the contig-level genome (Fig. 2).

### RNA library construction and transcriptome sequencing

Total RNA was extracted from five tissues of the live sharksucker, including dorsal muscle, dorsal fin, skin, skull, and skull muscle using TRIzol reagent (Invitrogen). To assess RNA quality, both a NanoDrop ND-1000 spectrophotometer (Labtech) and a 2100 Bioanalyzer (Agilent Technologies) were employed. The paired-end raw sequencing was performed using the Novaseq 6000 Platform. In sum, 33.01 Gb of clean data were generated from the RNA-seq library after filtering process using fastp v 0.23.2^16^ with default parameters (Table 1).

### Repetitive sequence annotation

**Fig. 4:**
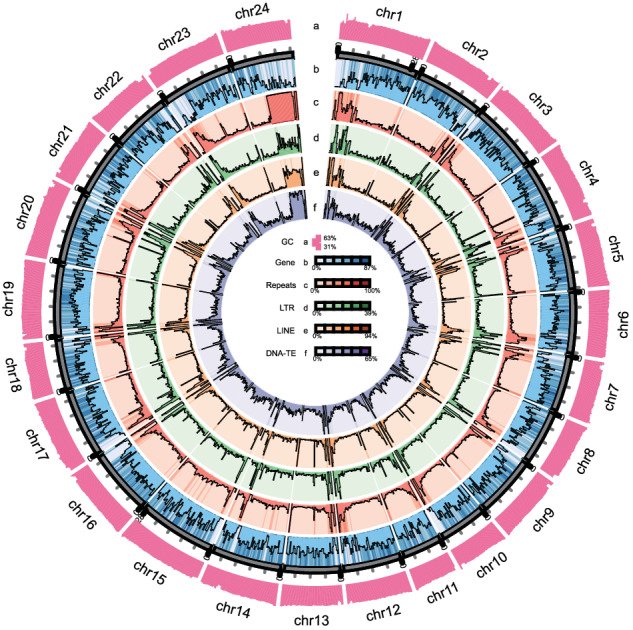
Chromosome-level assembly genomic landscape of *E. naucrates*. Circos plot from the outer to the inner layers represents the following: (**a**) GC content (range: 31% - 63%); (**b**) gene density (range: 0% - 87%); (**c**) repeat density (range: 0% - 100%); (**d**) LTR retroelement density (range: 0% - 39%); (**e**) LINE density (range: 0% - 94%); and (**f**) DNA transposons density (range: 0% - 65%). **a**-**f** were drawn in 500-kb sliding windows, and y-axes represent the proportion of respective elements within the window.

Repeat elements within the live sharksucker genome were comprehensively identified through a dual approach involving both homology searches and *ab initio* predictions. The *ab initio* prediction of repeat elements was executed using both Tandem Repeat Finder v 4.09^23^ and LTR_FINDER_parallel v1.1^23^ with default parameters. Subsequently, novel repeats were predicted utilizing RepeatMasker according to the de novo repetitive sequence library constructed with LTR_FINDER_parallel and RepeatModeler v 2.0^24^ following default parameters. To identify known repeat elements for genome sequences, RepeatMasker v 4.0.9^25^ and RepeatProteinMask v 4.1.0 (http://www.repeatmasker.org) with default parameters were employed, by querying the genome sequences against the Repbase database^26^. The integration of *ab initio* predictions and Repbase-based searches unveiled that 15.57% of the assembled *E. naucrates* genome was identified as repetitive sequences (Fig. 4). Among which, repetitive DNAs, LINEs, SINEs and LTRs covered 5.74%, 4.03%, 2.27% and 1.85% of the entire genome, respectively (Table 3).

**Table 3:** Statistics on transposable elements in *E. naucrates* genome.

Type	Repbase TEs		TE protiens		De novo		Combined TEs	
	Length (Bp)	% in genome	Length (Bp)	% in genome	Length (Bp)	% in genome	Length (Bp)	% in genome
DNA	23,226,811	3.95	4,315,166	0.73	17,014,011	2.89	33,789,962	5.74
LINE	15,566,251	2.65	10,793,779	1.83	13,058,906	2.22	23,708,510	4.03
SINE	4,469,188	0.76	0	0	9,252,845	1.57	13,382,324	2.27
LTR	7,628,300	1.3	3,715,461	0.63	4,045,882	0.69	10,854,685	1.85
Satellite	1,618,664	0.28	0	0	1,282,596	0.22	2,628,493	0.45
Simple_repeat	0	0	0	0	28,950	0	28,950	0
Other	4,276	0	111	0	0	0	4,387	0
Unknown	421,320	0.07	11,613	0	14,599,342	2.48	14,888,000	2.53
Total	48,853,610	8.3	18,810,305	3.2	57,392,278	9.76	91,569,318	15.57

### Gene prediction and annotation

Using the repeat-masked genome, the prediction of protein-coding genes within the live sharksucker genome was approached through three strategies: *ab initio* predictions, homologous searches and RNA-sequencing methods. *Ab initio* prediction was conducted utilizing Augustus v 3.3.2^27^ and Genscan^28^ tools with default parameters. In parallel, homologous gene prediction was based on the retrieval of protein sequences from various species, comprising *Caranx melampygus* (GenBank assembly accession: GCA_019059645.1^29,30^), *Echeneis naucrates* (GenBank assembly accession: GCA_900963305.1^13,14^), *Danio rerio* (GenBank assembly accession: GCA_000002035.4^31^), *Seriola dumerili* (GenBank assembly accession: GCA_002260705.1^32,33^), *Takifugu rubripes* (GenBank assembly accession: GCA_901000725.3^34^), and *Seriola lalandi* (GenBank assembly accession: GCA_002814215.1^35,36^). These protein sequences were downloaded from the NCBI database and subjected to alignment with our live sharksucker genome via tBLASTn (E-value ≤ 1e-5). Subsequently, the homologous genome sequences were aligned with the corresponding proteins through the utilization of Genewise v 2.4.0^37^ to obtain precise gene annotation. A pooled RNA-seq dataset of five tissues, each sequenced separately, were aligned to the assembled genome utilizing HISAT2 v 2.1.0^38^ with default parameters, and subsequently the putative transcript structures were predicted using StringTie v1.3.5^39^ and TransDecoder v 5.1.0 (https://github.com/TransDecoder/TransDecoder) with default parameters. Three gene models underwent merging to eliminate redundancy using MAKER v 2.31.10^40^ and HiFAP (Wuhan OneMore Tech Co., Ltd., https://www.onemore-tech.com/) with default parameters, resulting in the identification of 22,161 and 22,086 genes, respectively (Fig. 4 and Table 4).

**Table 4:** Statistics of gene predictions in the *E. naucrates* genome.

	Gene set	Number	Average gene length (bp)	Average CDS length (bp)	Average exon per gene	Average exon length (bp)	Average intron length (bp)
denovo	Genscan	27,258	14,277.38	1,660.71	9.36	177.48	1,509.65
	AUGUSTUS	24,750	10,233.76	1,476.47	8.42	175.38	1,180.43
Homolog	Caranx_melampygus	37,229	9,333.59	1,259.58	7.61	165.46	1,221.00
	Echeneis_naucrates_ncbi	30,043	11,329.89	1,596.51	8.92	178.96	1,228.77
	Danio_rerio	32,853	11,344.86	1,392.15	7.77	179.07	1,469.17
	Seriola_dumerili	30,681	11,034.62	1,517.16	8.54	177.73	1,262.87
	Takifugu_rubripes	29,116	10,480.93	1,497.16	8.55	175.11	1,189.91
	Seriola_lalandi	33,340	10,637.15	1,477.77	8.24	179.45	1,265.96
RNA-seq	Trans.orf	4,478	12,146.34	1,380.29	9.48	190.79	1,219.70
BUSCO		3,645	9,567.93	1,807.94	11.87	152.35	714.07
MAKER		22,161	12,728.30	1,742.10	10.08	183.00	1,198.85
HiCESAP		22,086	11,860.56	1,756.47	10.19	182.11	1,088.42

The predicted protein-coding gene sets were functionally annotated based on NCBI nonredundant protein (NR), Swiss-Prot^41^ (http://www.gpmaw.com/html/swiss-prot.html), TrEMBL (http://www.uniprot.org), eukaryotic orthologous groups of proteins (KOG)^42^, AnimalTFDB v4.0 (http://bioinfo.life.hust.edu.cn/AnimalTFDB4/?#/), and Kyoto Encyclopedia of Genes and Genomes (KEGG) (http://www.genome.jp/kegg/) using BLASTp^43^ (E-value ≤ 1e-5). The annotation of gene sets compared with InterPro and Pfam databases were performed via InterProScan v 5.61^44^ with parameters “--goterms–pathways--dp”. Finally, 21,402 genes (representing roughly 96.90% of total predicted genes) were effectively annotated by at least one of these databases (Table 5).

**Table 5:** Summary of functional annotations for predicted genes.

		Number	Percent (%)
Total		22,086	
Annotated	Merged	21,402	96.9
	InterPro	19,595	88.72
	GO	15,016	67.99
	KEGG_ALL	21,085	95.47
	KEGG_KO	13,863	62.77
	Swissprot	19,133	86.63
	TrEMBL	21,196	95.97
	TF	3,388	15.34
	Pfam	18,885	85.51
	NR	21,345	96.64
	KOG	17,722	80.24
Unannotated		684	3.1

### Non-coding RNA prediction and annotation

The ribosomal RNAs (rRNAs), microRNAs (mRNAs) and small nuclear RNAs (snRNAs) were predicted by using INFERNAL v.1.1^45^ according to the rfam^46^ and miRBase^47^ databases. Transfer RNAs (tRNAs) were annotated with tRNAscan-SE v 1.3.1^48^ following the default parameters. Taken together, non-coding RNAs, comprising 2,107 rRNAs, 1,786 miRNAs, 1,408 snRNAs and 12,200 tRNAs were predicted from the *E. naucrates* genome (Table 6).

**Table 6:** Statistics of ncRNA in *E. naucrates* genome.

Type		Copy	Average length(bp)	Total length(bp)	% of genome
miRNA		1,786	88	157,430	0.027
tRNA		12,200	76	924,168	0.157
rRNA	rRNA	2,107	193	407,311	0.069
	18 S	92	1,789	164,554	0.028
	28 S	0	0	0	0.000
	5.8 S	90	154	13,859	0.002
	5 S	1,925	119	228,898	0.039
snRNA	snRNA	1,408	146	204,979	0.035
	CD-box	139	116	16,173	0.003
	HACA-box	61	151	9,213	0.002
	splicing	1,202	148	178,147	0.030
	scaRNA	6	241	1,446	0.000

### Identification of telomeres

Based on the common characteristic sequences (CCCTAA/TTAGGG) of vertebrates, telomere sequences are identified through pattern searching at both ends of each chromosome, where the characteristic sequence repeats at least four times within a 50 kb region. All 38 telomeres were annotated within the 23 chromosomes, with no telomere sequence detected on chr7 (Fig. 3a and Table 7).

**Table 7:** Telomeres in *E. naucrates* genome.

Chr ID	Number of start telomere repeats	Number of end telomere repeats
chr1	0	835
chr2	1254	954
chr3	0	1692
chr4	851	0
chr5	1005	1343
chr6	75	0
chr7	0	0
chr8	1340	1567
chr9	1113	1356
chr10	808	1533
chr11	0	1146
chr12	1661	1412
chr13	1346	16
chr14	842	957
chr15	1004	1696
chr16	60	1162
chr17	0	699
chr18	1899	1070
chr19	269	884
chr20	1473	0
chr21	1572	107
chr22	1728	0
chr23	1186	98
chr24	1186	1195

## Data Records

The raw sequencing dataset of *E. naucrates* in this study can be achieved from Sequence Read Archive (SRA) under SRP457893^49^, including WGS Illumina sequencing data (SRR25859131), Pacbio HiFi sequencing data (SRR25859130) and Hi-C sequencing data (SRR25859129). The assembled genome of *E. naucrates* was deposited at GenBank under accession GCA_031770045.1^50^. Furthermore, files of the assembled genome, protein-coding gene annotation, non-coding RNA prediction and repeat annotation of *E. naucrates* were deposited in Figshare database^51^.

## Technical Validation

### Assessing the quality of the genome assembly

We initially used QUAST v 5.2.0^52^ to evaluate the integrity and quality of *E. naucrates* genome assembly. The contig N50 (the length at which half of the total sequence resides in contigs of this size) has shown a significant improvement, reaching 23.19 Mb, which significantly surpasses previous *E. naucrates* genome versions of 12.4 Mb (GenBank assembly accession: GCA_900963305.1, GCA_900963305.2). Furthermore, in this study, the genome exhibits an exceptionally low gap count (average 0.40 N’s per 100 kbp) (Table 2; Fig. 3a), marking a substantial reduction compared to the previous versions of average 110.13 N’s per 100 kbp (Fig. 3b). Next, we remapped Illumina paired-end clean reads and PacBio long reads to the final assembled genome using BWA^53^ and Minimap2^54^, resulting in mapping rates of 99.62% and 99.98%, respectively. Homozygous SNP rate was 0.00% when aligned Illumina paired-end clean reads to the final assembly, underscoring the comprehensiveness of the complete genome (Table 8). Furthermore, the completeness of the assembled genome sequence was assessed with Benchmarking Universal Single-Copy Orthologs (BUSCO, v 5.1.0)^55^ based on the actinopterygii_odb10 database. The BUSCO analysis of assembly showed that 3,551 (97.5%) of the complete orthologs, including 3,514 (96.5%) single-copy orthologs and 37 (1.0%) duplicated orthologs, as well as 14 (0.4%) fragmented orthologs were identified (Table 9). The consensus quality value (QV) of the assembly, estimated using Merqury^56^ (kmer = 21), was 52.01.

**Table 8:** Statistics of *E. naucrates* SNPs and InDels.

Type	Percentage (%)
Homozygous SNP	0
Homozygous InDel	0.001
Heterozygous SNP	0.253
Heterozygous InDel	0.09

**Table 9:** Statistics of BUSCO assessment.

	Proteins	Assembly	Proteins	Annotation
		Percentage (%)		Percentage (%)
Complete BUSCOs	3,551	97.5	3,473	95.4
Complete Single-Copy BUSCOs	3,514	96.5	3,437	94.4
Complete Duplicated BUSCOs	37	1	36	1
Fragmented BUSCOs	14	0.4	46	1.3
Missing BUSCOs	75	2.1	121	3.3
Total BUSCO groups searched	3,640	100	3,640	100

### Assessing the quality of the genome annotation

The BUSCO analysis of annotation based on the actinopterygii_odb10 database, which was used to assess the integrity of the annotated gene set, revealed that 95.4% (3,473) of the complete genes were identified, comprising 94.4% (3,437) single-copy genes, 1.0% (36) duplicated genes, and 1.3% (46) fragmented genes (Table 9).

Taken together, the comprehensive assessment of the *E. naucrates* genome surpassed that of other existing public *E. naucrates* genomes.

## Data Availability

No specifc code was used in this study. The data analyses adhered to the manuals and protocols offered by the creators of the corresponding bioinformatics tools, the parameter settings of which were outlined in the methods section.
